# Methods to Improve Osseointegration of Dental Implants in Low Quality (Type-IV) Bone: An Overview

**DOI:** 10.3390/jfb9010007

**Published:** 2018-01-13

**Authors:** Hamdan S. Alghamdi

**Affiliations:** Department of Periodontics and Community Dentistry, College of Dentistry, King Saud University, Riyadh 11545, Saudi Arabia; dalghamdi@ksu.edu.sa; Tel.: +966-11-467-7732

**Keywords:** dental implants, osseointegration, bone regeneration, surface modifications

## Abstract

Nowadays, dental implants have become more common treatment for replacing missing teeth and aim to improve chewing efficiency, physical health, and esthetics. The favorable clinical performance of dental implants has been attributed to their firm osseointegration, as introduced by Brånemark in 1965. Although the survival rate of dental implants over a 10-year observation has been reported to be higher than 90% in totally edentulous jaws, the clinical outcome of implant treatment is challenged in compromised (bone) conditions, as are frequently present in elderly people. The biomechanical characteristics of bone in aged patients do not offer proper stability to implants, being similar to type-IV bone (Lekholm & Zarb classification), in which a decreased clinical fixation of implants has been clearly demonstrated. However, the search for improved osseointegration has continued forward for the new evolution of modern dental implants. This represents a continuum of developments spanning more than 20 years of research on implant related-factors including surgical techniques, implant design, and surface properties. The methods to enhance osseointegration of dental implants in low quality (type-IV) bone are described in a general manner in this review.

## 1. Introduction

Dental implants have become a more common treatment for replacing missing teeth [1]. Consequently, in clinical dentistry, dental implants aim to increase patient satisfaction in terms of improved chewing efficiency, physical health, and esthetics. The global dental implant market is anticipated to grow steadily from US$3.4 billion in 2011 to US$6.4 billion in 2018 [1]. The favorable clinical performance of dental implants has been attributed to their firm bone integration.

In 1965, Brånemark introduced the term “osseointegration” to describe the successful outcome of bone-to-implant integration [2]. Clinically, the process of osseointegration reflects the mechanical anchorage of a dental implant into the jaw bone that persists under all normal conditions of oral function. Overall, bone regeneration related to dental implants in a healthy condition is a complex process and can take up to several weeks. A few days after implantation, several biological events (bone regeneration) are regulated by several growth and differentiation factors that are released in the implant vicinity [3,4]. The process of bone regeneration is formed either on the implant surface (i.e., de novo bone formation, contact osteogenesis) or from the surrounding bone towards the implant surface (i.e., distance osteogenesis) [5]. Finally, bone remodeling occurs by replacing immature with mature bone at the implant site, providing biological (mechanical) stability, which is secondary to primary fixation obtained during implant insertion.

**Figure 1:**
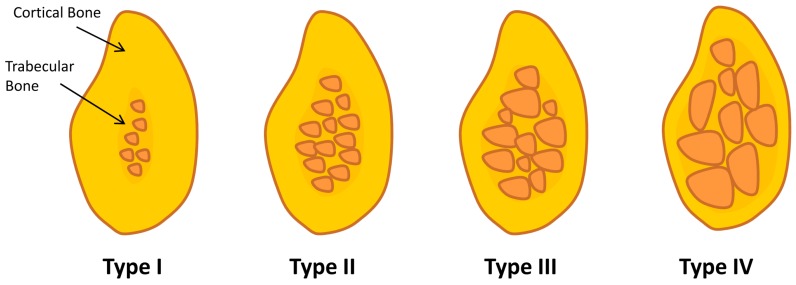
Lekholm & Zarb classification: Type I, the entire bone is composed of very thick cortical bone; Type II, thick layer of cortical bone surrounds a core of dense trabecular bone; Type III, thin layer of cortical bone surrounds a core of trabecular bone of good strength; and Type IV, very thin layer of cortical bone with low density trabecular bone of poor strength.

Although the survival rate of dental implants over a 10-year observation has been reported to be higher than 90% in totally edentulous jaws [6], dental implants do fail in some patients. There are many reasons for dental implant failure including an inappropriate diagnosis and treatment plan, inadequate information on the patient’s medical history, or lack of experience and surgical skills to place dental implants correctly [7]. Most importantly, the clinical outcome of implant treatment is challenged in compromised (bone) conditions, as are frequently present in elderly patients. For example, epidemiological data show that osteoporosis is increasing among the elderly female and male (>65 years) population [8,9]. A striking characteristic of the osteoporotic condition is the severe reduction of bone quality and quantity which is suggested to be detrimental for bone–implant integration [9]. Additionally, the biomechanical characteristics of osteoporotic bone do not offer proper stability to implants, being similar to type-IV bone (Lekholm & Zarb classification; Figure 1), in which a decreased clinical fixation of implants has been clearly demonstrated [7,10]. Thus, decreased osteogenic capacity of bone in an osteoporotic condition can be considered as a possible risk factor for implant failure. Such a risk of implant failure in osteoporotic bone is hypothetically related to various factors that compromise bone–implant healing and potentially impair osseointegration. An impairment of bone–implant regeneration in osteoporotic condition includes the imbalanced activity of osteoblast/osteoclast cells acting on bone formation and remodeling. In addition, proliferation and activity of mesenchymal cells and osteoblastogenesis seem affected [11]. Although research on osteoporosis is ongoing, including prevention and treatment modalities, the knowledge on bone-biomaterial regeneration in osteoporotic bone remains limited. However, the search for improved bone regeneration in challenged conditions has helped propel the continuing evolution of modern dental implants. This represents a continuum of developments spanning more than 20 years of research on implant related-factors including surgical techniques, implant design, and material and surface properties.

To explain the above-mentioned problem better, the remedies to enhance bone–implant integration in low quality (Type IV) bone are described in a general manner in this review. 

## 2. Design of Dental Implants and Primary Stability

**Figure 2:**
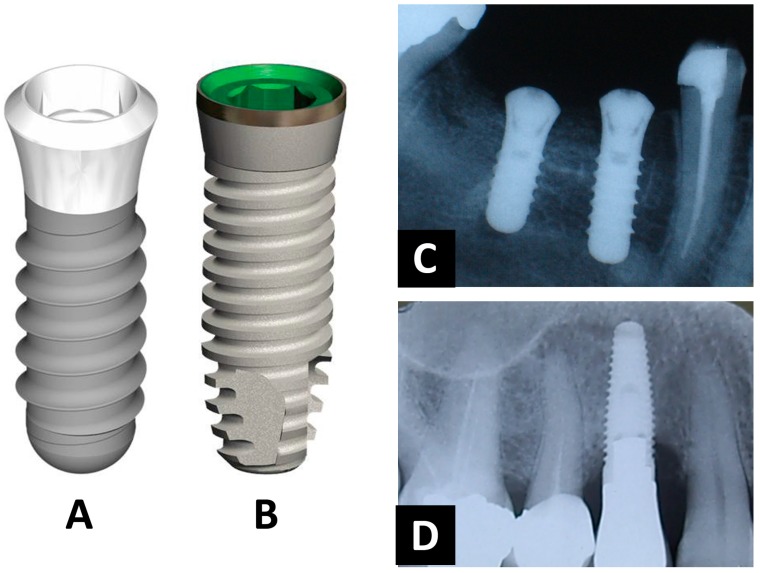
Dental implants are mostly available as (**A**) threaded cylindrical-shaped or (**B**) conical (root)-shaped (Images are courtesy of Baltic Osseointegration Academy/Public Information. http://www.boaoffice.lt/EN/9/). Radiographic images displaying clinical cases in which both ((**C**,**D**), respectively) implant-designs are inserted in the jaw bone.

Over the last few decades, several implant-design concepts have been developed and are commercially available. Dental implants mostly possess a threaded cylindrical- or conical(root)-design (Figure 2). The design parameters primarily affect load (i.e., stress/strain) distribution in the bone tissue, resulting in a proper implant fixation and function. These include implant diameter and length as well as thread pitch, shape, and depth. Additionally, the presence of threads increases the surface area for osseointegration, and thereby aids in the achievement and maintenance of direct bone–implant integration [12]. Implant geometry can significantly enhance initial stability and the biomechanical fixation of the implant after the healing process [13]. Therefore, dental implant stability is a prerequisite parameter to promote the process of osseointegration, consequently ensuring the success of an implant treatment [13]. Depending on bone quantity and quality, the degree of implant anchorage may also be affected directly by the condition of bone itself as well as the stiffness of the implant-bone interface. However, it has to be mentioned that the relationship between the design of an implant and its primary stability is still subject to controversy in literature [13].

Despite an important role of biomechanics in the establishment of osseointegration, implant-design concepts are not expected to promote advantageous functions by specific control of bone cells and tissues interactions at the implant interfaces.

## 3. The Importance in Surgical-Implantation Techniques

Conventionally, the placement of dental implants sacrifices much bone tissue during the drilling procedure, which is performed with a consecutive series of surgical drills to prepare an implant bed fits the implant exactly. Consequently, several modalities of implantation techniques have been proposed to optimize a high degree of implant stability without removing additional bone, especially in situations where limited bone density (i.e., challenged condition) is available. For example, a surgical technique has been introduced that compresses the bone tissue laterally and apically using an osteotome spreader [14]. Additionally, the ‘undersized drilling’ technique has been also explored extensively and most implant manufacturers are currently recommending the undersized drilling technique for implant placement [15]. In this procedure, bone density is locally optimized by lateral bone compression along the implant sides using a final drill diameter considerably smaller than the implant diameter. This method has resulted in higher insertion torque values, which, in turn, are the indicator of improved primary implant (mechanical) stability [15]. Besides enhancing the primary stability of an implant, the undersized surgical technique showed the additional advantage of osteogenic bone fragments becoming translocated and interspersed along the surface of the implant, with evident signs of contribution of these bone particles to stimulate peri-implant bone healing and remodeling [16]. Still, further studies should be performed to evaluate the biological mechanisms underlining the favorable results of the undersized drilling technique and its beneficial role in the process of new bone formation.

## 4. Physicochemical Surface Modifications for Dental Implants

**Figure 3:**
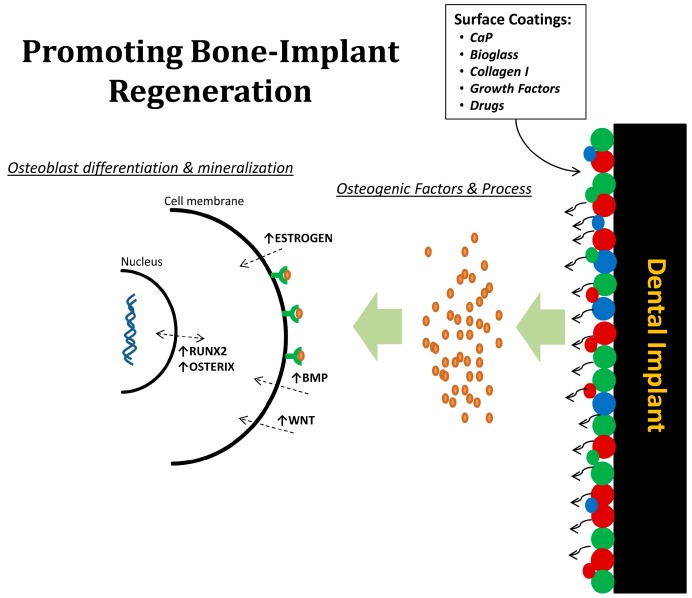
Illustration representing biological beneficial of surface coatings on dental implants. Drugs or bioactive factors can be delivered to the local bone microenvironment around implants, in which osteogenic factors and process are promoted; thereby increasing osteoblast differentiation and mineralization.

The osseointegration process relates to the all biological interactions between the host bone and implant surface. In view of this, implant surface modification is considered as an important approach to favor this process (Figure 3). Implant surface modification enhances the interactions with biological fluids and cells and accelerates peri-implant bone healing as well as improves osseointegration at sites that lack sufficient quantity or quality of bone [4]. In the past two decades, various surface modification approaches have been proposed and studied to improve implant osseointegration. For instance, implant surface micro-roughness offers an advantage as the area of contact is enlarged, which plays a significant role in anchoring cells and connecting to surrounding tissues, thereby favoring peri-implant osteogenesis [17]. Different methods have been developed to modify implant surface micro-roughness, of which grit-blasting, acid etching, or combinations are most commonly used. Grit-blasting is performed by projection of silica (sand-blasting), hydroxyapatite, alumina, or titanium oxide (TiO_2_) particles, and is followed commonly by acid-etching to homogenize the micro-profile of the implant surface and to remove as much of the residual blasting particles as possible. Acid-etching is often performed using hydrofluoric, nitric, sulfuric acid or combinations thereof [18]. Recently, the modification of the implant surface at the nanoscale level has been also introduced, which is based on the assumption that mimicry of the nano-pattern of bone structures might increase the surface energy—and hence improve matrix protein adsorption, bone cell migration, and proliferation—and finally enhance osseointegration [19]. However, further investigations are needed to explore the capacity of nanometer scale surface topographies to enhance the osteogenicity of titanium implants.

In contrast to the physical modifications, the deposition of bioactive coatings onto the implant surface by means of several biochemical deposition techniques have been explored [20,21], among which calcium phosphate (CaP) coatings have received significant attention because of their chemical similarity to the natural bone mineral, and the fact that coatings can be applied along the implant surfaces by different industrial processing methods [20]. CaP-based implant coatings show the ability to directly bond to bone tissue and increase the biochemical interlocking between bone and surface materials [20]. Other efforts focused on the deposition of bioactive molecules, such as extracellular matrix (ECM) proteins collagen, enzymes, and growth factors. Coating titanium surfaces with ECM-proteins are found to enhance implant osseointegration through the accelerated speed and amount of new bone formed at the interface [21]. More recently, in vitro and in vivo investigations have suggested that implant surface coatings combining CaP ceramics and bioactive molecules might play an instructive (i.e., osteoconductive) role to control peri-implant osteogenesis, and thereby accelerate the process of bone–implant interactions [22]. In an animal study, we hypothesized that coating of titanium implant surface with CaP or collagen type-I can significantly improve the implant-bone response in osteoporotic and healthy conditions. Interestingly, CaP and collagen type-I surface coatings enhanced implant osseointegration by 1.6- to 2.0-fold compared to control groups. In this study, it was confirmed that the use of osteogenic surface coatings has a favorable effect on the bone–implant interface in both osteoporotic and healthy conditions [23]. In addition to CaP and collagen type-I, growth factors like bone morphogenetic proteins (BMPs) and transforming growth factor-β1 (TGF-β1) have also been investigated for accelerating bone regeneration around dental implants [24]. For instance, Lan et al. [25] and co-workers evaluated the influence of rhBMP-2 on implant osseointegration in a rabbit animal model. Their results showed a positive effect of rhBMP-2 on the quantity and quality of bone formation around implants. However, other preclinical studies reported no beneficial effect of implant surface coatings with growth factors on osseointegration [26,27].

## 5. Drug-Based Implants Modification

Additionally, new coating strategies to improve implant osseointegration involve the development of a dedicated drug-loading ability to locally target bone disorders around dental implants more effectively [28]. For instance, antiresorptive (e.g., bisphosphonates) and anabolic (e.g., strontium ranelate and statins) agents might improve implant osseointegration in osteoporotic bone [29]. When these pharmacological drugs are incorporated onto the surface of implant, and released gradually and locally in the peri-implant area, the bone healing process might be improved. Recently, we used an implantation model in osteoporotic and healthy animals to analyze titanium implants coated with bisphosphonate (BP)-loaded calcium phosphate nanoparticles (nCaP). After four weeks of implantation, bone regeneration was significantly increased around the implant surfaces as functionalized with BP. The results of osteogenic gene expression were similar to all implant surfaces. In conclusion, using nCaP/BP surface coatings represents an effective strategy for improving bone–implant integration, especially in compromised bone conditions [30].

From the aforementioned findings, it appears that there are many methods to modify implant performance by improving the bone response physically, chemically, or therapeutically. However, the exact underlying biological mechanisms of these methods have not been fully characterized. Consequently, research has to focus on the in vivo investigation of existing implant surface modifications in order to achieve the desired biological responses, especially in compromised conditions. Long-term in vivo preclinical research is also necessary to investigate instructive as well as therapeutic capabilities of an implant surface coating using osteoporotic animal models.

## 6. Experimental Models for Dental Implants

For the investigation of the osseointegration of bone implants with a newly developed surface modification, animal experiments are of fundamental significance. Several animal models are commonly used to study osseointegration (i.e., peri-implant osteogenesis) [31]. However, to explore the biological efficacy of an implant surface designed to be applied in a compromised health condition, a specific animal model is needed resembling the medical condition being investigated which is capable of demonstrating a relevant biological response prior to clinical use. For instance, models reproducing an osteoporotic condition can be useful to help understand the influence of the osteoporotic pathology on implant osseointegration. Induction of an osteoporotic condition has been proposed using different methods, including the surgical removal of gonadal tissues, which leads to the development of a systemic osteoporotic condition in both the peripheral and axial skeleton of animal. This might experimentally mimic the complex bone–implant interactions and the decrease in osteoinductive capacity that accompany osteoporotic conditions in humans [32,33]. Irrespective of the animal models, valuable information can be retrieved from properly designed in vivo experiments. Static and dynamic histomorphometrical and radiographical examinations as well as biomechanical testing are recommended to evaluate peri-implant osteogenesis where different surface modifications are compared. For instance, peri-implant bone contact and amount are commonly evaluated in vivo, and are examples of static parameters. Differently, fluorescence analysis provides valuable dynamic measurements of the bone healing around implant surfaces. Finally, for an accurate judgment of the obtained results regarding peri-implant osteogenesis, an in vivo experimental setup should be well designed and statistical analysis should be well conducted [34].

## 7. Conclusions

The developments regarding implant surface modifications seem critical for bone healing and improving osseointegration at sites that lack sufficient quantity or quality of bone. Thus, the research efforts at present are attempting to overcome the problems associated with implant complications that might arise in challenged bone conditions through the development of the surface-coated bone implants. Additionally, in vivo experiments that successfully addressed the instructive/therapeutic capabilities of implant surface coatings should be continued.
